# Clinic Attendances during the First 12 Months of Life for Aboriginal Children in Five Remote Communities of Northern Australia

**DOI:** 10.1371/journal.pone.0058231

**Published:** 2013-03-01

**Authors:** Thérèse Kearns, Danielle Clucas, Christine Connors, Bart J. Currie, Jonathan R. Carapetis, Ross M. Andrews

**Affiliations:** 1 Child Health Division, Menzies School of Health Research, Charles Darwin University, Darwin, Northern Territory, Australia; 2 Department of Paediatrics, The University of Melbourne, Melbourne, Victoria, Australia; 3 Preventable Chronic Conditions, Northern Territory Department of Health and Families, Darwin, Northern Territory, Australia; 4 Telethon Institute for Child Health Research, Centre for Child Health Research, University of Western, Perth, Western Australia, Australia; The Australian National University, Australia

## Abstract

**Background:**

The vast majority (>75%) of Aboriginal people in the Northern Territory (NT) live in remote or very remote locations. Children in these communities have high attendance rates at local Primary Health Care (PHC) centres but there is a paucity of studies documenting the reason and frequency of attendance. Such data can be used to help guide public health policy and practice.

**Methods and Findings:**

Clinic presentations during the first year of life were reviewed for 320 children born from 1 January 2001–31 December 2006. Data collected included reason for infectious presentation, antibiotic prescription and referral to hospital. The median number of presentations per child in the first year of life was 21 (IQR 15–29) with multiple reasons for presentation. The most prominent infectious presentations per child during the first year of life were upper respiratory tract infections (median 6, IQR 3–10 ); diarrhoea (median 3, IQR 1–5); ear disease (median 3, IQR 1–5); lower respiratory tract infection (median 3, IQR 2–5); scabies (median 3, IQR 1–5); and skin sores (median 3, IQR 2–5).

**Conclusions:**

Infectious diseases of childhood are strongly linked with poverty, poor living conditions and overcrowding. The data reported in our study were collected through manual review, however many remote communities now have established electronic health record systems, use the Key Performance Indicator System and are engaged in CQI (continuous quality improvement) processes. Building on these recent initiatives, there is an opportunity to incorporate routine monitoring of a range of infectious conditions (we suggest diarrhoea, LRTI, scabies and skin sores) using both the age at first presentation and the median number of presentations per child during the first year of life as potential indicators of progress in addressing health inequities in remote communities.

## Introduction

Disparities in disease burden and life expectancy for Aboriginal and Torres Strait Islander people in Australia are well documented, even more so for those living in remote/very remote locations. [1] In the Northern Territory (NT), more than 75% of Aboriginal people live in remote or very remote locations. [1] Previously reported data from three remote communities highlighted very high clinic attendance rates and a previously unrecognized burden of skin infections commencing within the first few months of life. [2], [3] We have now extended the study to include six birth cohorts (2001–2006) across five remote Aboriginal communities within the same region. We have focused our analyses using the presentations per child as the primary unit of measure and report clinic presentations for a range of infectious conditions.

## Methods

We reviewed clinic presentations during the first year of life for children in five remote communities born from 1 January 2001 to 31 December 2006. The communities, located approximately 500–1000 km east of Darwin, were participating in the East Arnhem Regional Healthy Skin Project (EARHSP). [4] Children were recruited to the EARHSP by local researchers going house to house. Written informed consent was obtained from the parents/guardian to screen their child for clinical signs of scabies and skin sores and to conduct an audit of their clinic presentations.

Each community had a locally based Primary Health Care (PHC) centre, providing acute and chronic care as well as an after-hours emergency service. We estimated the total annual birth cohort for these five communities from 2001–2006 to be approximately 1500 (250 per year). For the purposes of this study, data for 320 children were included from a total of 450 from whom consent had been obtained. To be included in this study children had to have been seen at the PHC at least once in each age bracket of 0-<3months, 3-<6months, 6-<9 months and 9-<12 months. Multiple presentations on the same day were recorded as the one presentation. Presentations with missing or incomplete dates were excluded.

We utilized the same methods for review of presentations to PHC services as previously described. [2] We manually reviewed the clinic presentation records for each child and recorded: the date of each presentation, the child’s height and weight, any infectious reason for presentation, antibiotic prescription and any referral to hospital. Each of the six communities had a designated clinic as the sole PHC service. One community also had a regional hospital nearby (within 20 km).

Ethics approval was obtained from The Human Research Ethics Committee of NT Department of Health and Families and Menzies School of Health Research (HREC ref 04/11).

Data were analysed in Stata version 12.1. [5] Data were examined per child and per presentation per child. Continuous skewed data were expressed as medians (interquartile range (IQR)) and dichotomous data as percentages. We assessed the contribution of scabies to the overall skin sore burden for presentations during the first year of life by calculating a risk ratio with 95% confidence interval (CI) of a diagnosis of skin sore infection at a presentation of scabies compared to no diagnosis of scabies at the same presentation. The method of generalized estimating equations was used with an exchangeable correlation structure in a log-binomial regression model to account for within-child correlation.

## Results

The 320 children in the birth cohort (1 January 2001–10 May 2006) had a total of 7272 presentations to the PHC service during their first year of life; 53% were male. The median number of presentations per child in the first year of life was 21 (IQR 15, 29). The median number of presentations per child was 7 (IQR 5–10) for non-infectious causes in the first year of life, including routine health checks, and was 14 (IQR 9–20) for infectious conditions.

**Figure 1:**
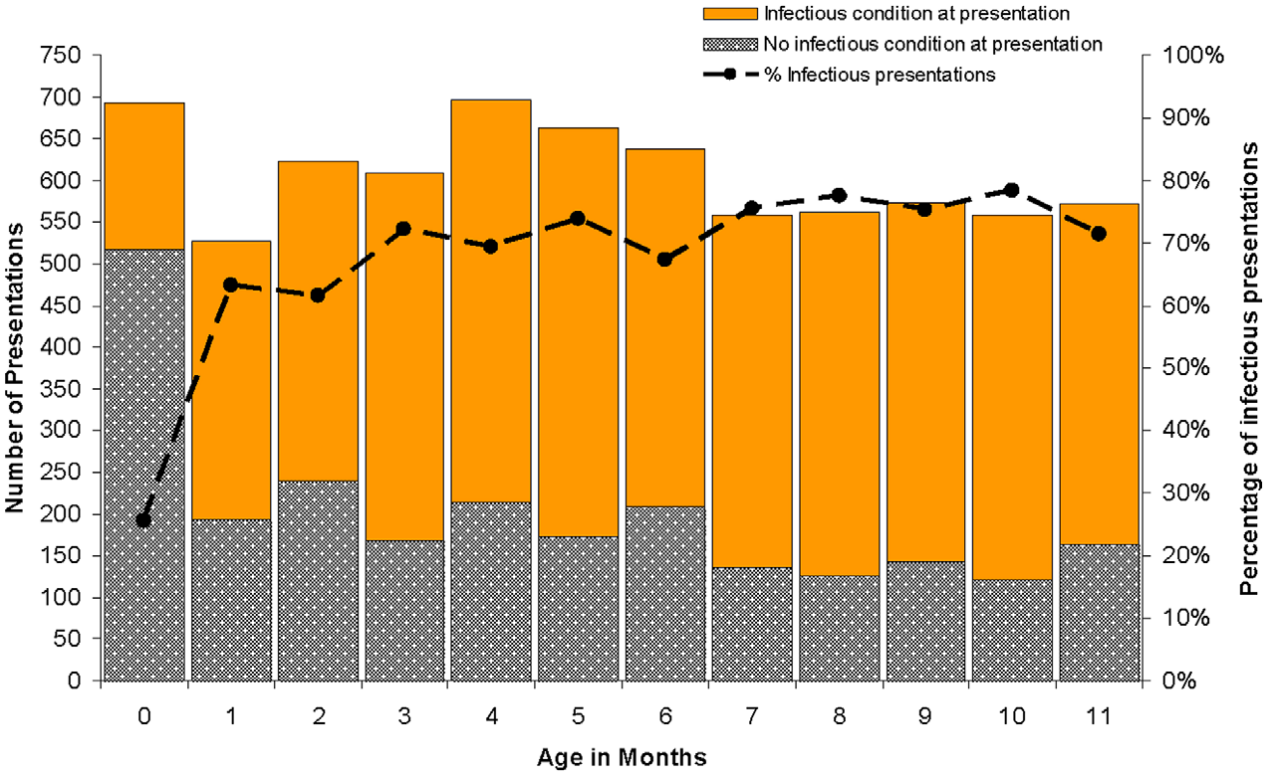
Presentations to health centre by age in months.

Other than the first month of life, almost two thirds of the presentations to the PHC service at each subsequent month of age included an infectious condition (Figure 1). There was a discernible but relatively moderate increase in presentations coded as being for non-infectious causes at 2, 4 and 6 months of age, coinciding with the childhood standard vaccination schedule.

As shown in Table 1, amongst our cohort the most prominent infectious presentations per child during the first year of life were: upper respiratory tract infection (median 6, IQR 3–10); diarrhoea (median 3, IQR 1–5); ear disease (median 3, IQR 1–5); lower respiratory tract infection (median 3, IQR 2–5); scabies (median 3, IQR 1–5); and skin sores (median 3, IQR 2–5). Overall, very few children managed to reach their first birthday without having at least one documented episode of an URTI (96%), scabies and/or skin sores (84%), ear disease (82%), diarrhoea (77%), or LRTI (75%). Recurrent presentations to the clinic for the same health condition were very common in a child’s first year of life; 285 children (92.8%) represented for URTI, 229 (85.1%) for scabies or skin sores, 191 (73.2%) for ear disease, 174 (70.5%) for diarrhoea and 181 (75.7) for LRTI.

**Table 1:** Reasons recorded for presentation at the health centre during the first year of life for a cohort of 320 children born from 1 Jan 2001–31 Dec 2006 and living in one of five remote Aboriginal communities in East Arnhem land.

Reason for presentation	Number of Children Presenting (%)^^^	Presentations per child median (IQR*)†	Children with more than one presentation n (%)#
**Upper respiratory tract infections (URTI)**	307 (95.9)^	6 (3, 10)	285 (92.8)
**Ear disease**	261 (81.6)^	3 (1, 5)	191 (73.2)
**Scabies and/or skin sores**	269 (84.1)^	4 (2, 7)	229 (71.6)
**Scabies**	221 (69.0)^	3 (1, 5)	156 (70.5)
**Skin Sores**	262 (81.9)^	3 (2, 5)	200 (76.3)
**Lower respiratory tract infections** **(LRTI)**	239 (74.7)^	3 (2, 5)	181 (75.7)
**Diarrhoea**	247 (77.2)^	3 (1, 5)	174 (70.5)
**Febrile Illness**	158 (49.4)^	2 (1, 4)	94 (59.5)
**Throat Infection**	88 (27.5)^	1 (1, 2)	27 (30.7)
**Tinea**	53 (16.6)^^^	1 (1, 2)	21 (39.6)
**Acute post-streptococcal glomerulonephritis**	0 (0.0)^	–	–
**Acute rheumatic fever**	1 (0.0)^	–	–
**Non-infectious cause**	319 (99.7)^	7 (5, 10)	311 (97.5)
**Total**	**320 (100)^**	**21 (15, 29)**	**320 (100)**

^The proportion of children presenting for a specified reason is equivalent to the cumulative incidence of that condition/reason during the first year of life.

* IQR = Interquartile range.

† Median number of presentations per child, per condition in the first year of life.

# % of children with coded as having the same reason for presentation (recurrence) in the first year of life.

**Figure 2:**
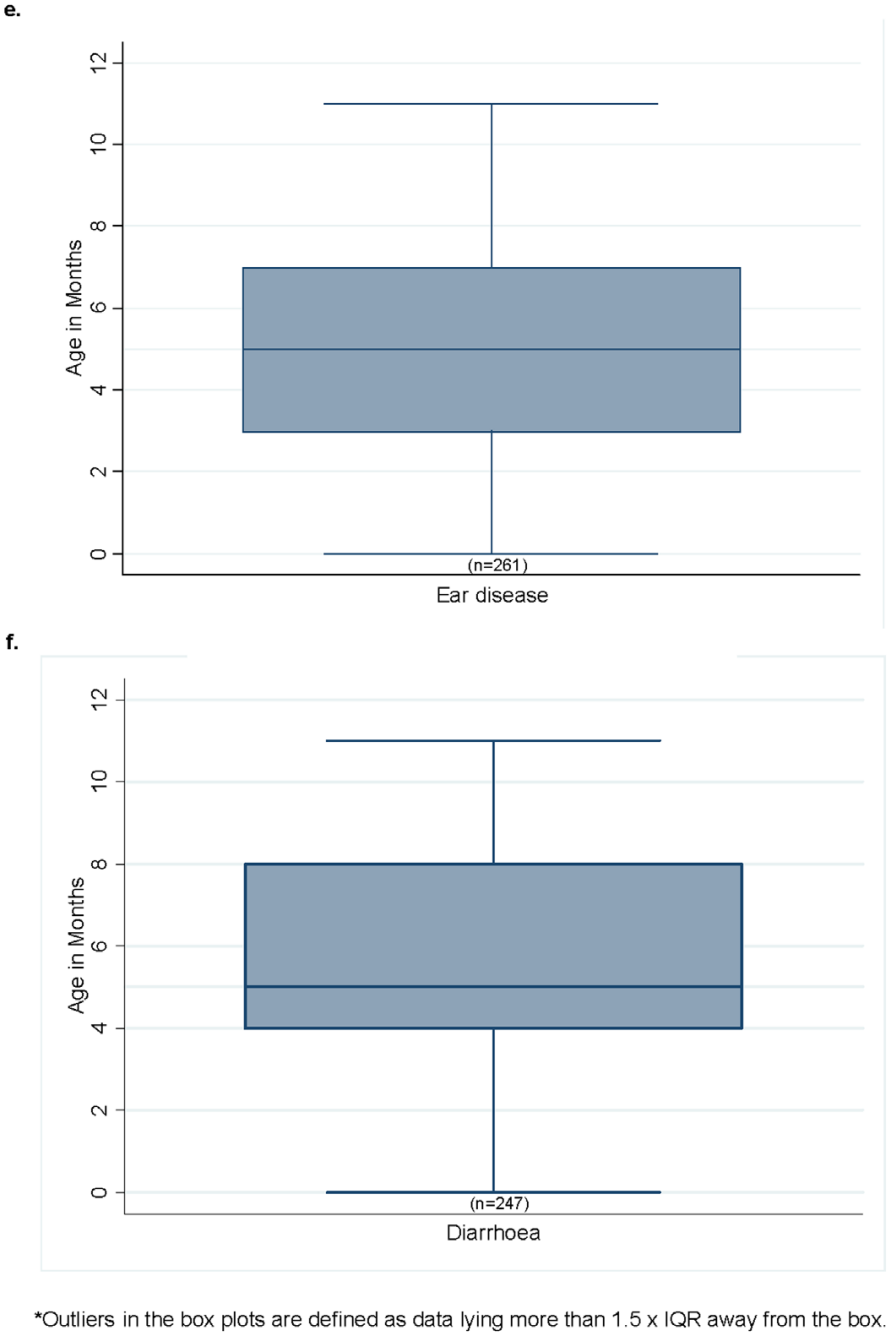
Median age at first clinic presentation for URTI, LRTI, scabies, skin sores, diarrhoea and ear disease in 5 remote communities in Northern Territory, Jan 01–Jan07.

The median age of the first presentation was 1 month (IQR 0–3) for URTI (Figure 2A), 4 months (IQR 2–6) for LRTI (Figure 2B), 4 months (IQR 2–7) for scabies (Figure 2C), 5 months (IQR 2–7) for skin sores (Figure 2D), 5 months (IQR 3–7) for ear disease (Figure 2E) and 5 months (IQR 4–8) for diarrhoea (Figure 2F).

Skin sores were 7.0 (95% CI 6.3, 8.0) times more likely to be concurrently diagnosed along with scabies than they were if there was not a scabies diagnosis.

## Discussion

Worldwide, the health disparity between Indigenous and non-Indigenous people is well recognized as is the heavy infectious disease burden. [6] Within Australia, the leading cause of hospitalization for children under 2 years of age is infection with Aboriginal children having a significantly higher admission rate than non-Aboriginal children. [7] Infectious diseases of childhood are strongly linked with poverty, poor living conditions and overcrowding. [8], [9].

Scabies is responsible for major morbidity worldwide as infestations facilitate skin sore infections by bacterial pathogens such as *Streptococcus pyogenes* and *Staphylococcus aureus*. [10] *Streptococcus pyogenes* can predispose children to secondary disease including rheumatic fever/heart disease which worldwide has the highest prevalence in Australian Aboriginal and Torres Strait Islander people. [11] In our study, the link between scabies and skin sore infections was illustrated by the finding that a child was 7.0 times more likely to have both scabies and skin sores than to have skin sores in the absence of scabies.

Within our cohort of 320 children, who were born from 2001–2006 and had spent the first year of their life in one of five remote Aboriginal communities in northern Australia, there were high rates of infectious disease presentations. We also observed frequent recurrences of the same infections among individual children. Upper respiratory tract infection was the most common with a median of 6 presentations per child in the first year of life. Diarrhoea, ear disease, lower respiratory tract infection, scabies and skin sores also predominated with a median of 3 presentations per child in the first year of life for each of these conditions. By one year of age 96% of the infants had attended the health service with at least one infectious episode.

Our findings support previously demonstrated high rates of clinic attendance in similar settings. [2], [3] We acknowledge that our study cohort of 320 children, 21% of the estimated birth cohort, may not be representative of all children born in these communities and particularly those children who did not remain resident for the first year. In regards to internal validity, all members of our cohort were required to have attended the clinic on at least one occasion in each of four sequential 3-month periods. We adopted these eligibility criteria in order to define the population at risk during the first year of life and used this is a proxy indicator of presence within the community over that time. We required the child to have had at least one visit to the PHC across a range of age strata which coincided with the expected contact visits for standard immunisations and represented the minimum number of visits a well child would have if resident in the community during the first year of life. Given high immunisation rates of 85% for children aged <1 year, [1] we considered this would not unduly lead to bias towards sick children. Moreover, high clinic attendance rates had been documented by us in this region (median 23 presentations during the first year of life). [2], [3] Of note, one third of presentations (a median of 7 per child) were for non-infectious causes, such as growth monitoring and immunisations, which again does not suggest a bias towards sick children.

The major strength of our study is in the utilization of existing datasets to monitor clinic presentations for infectious causes as a potentially useful indicator of progress towards improving health outcomes during the first of year of life. Many remote communities now have established electronic health record systems, use the Key Performance Indicator System and are engaged in CQI (continuous quality improvement) processes. Building on these recent initiatives, there is an opportunity to incorporate routine monitoring of a range of infectious conditions (we suggest diarrhoea, LRTI, scabies and skin sores) using both the age at first presentation and the median number of presentations per child during the first year of life as potential indicators of progress in addressing health inequities in remote communities. For example, monitoring changes in the median number of diarrhoeal presentations to Primary Health Care centres for a range of birth cohorts before and after the introduction of routine rotavirus vaccination could fill a gap in the assessments of vaccine effectiveness in the field. Monitoring clinic presentations in the first year of life for diarrhoea, LRTI, scabies and skin sores would be consistent with the Australian and Northern Territory Governments commitments to closing the gap between Indigenous and non-Indigenous Australians. [12].
